# *Saccharomyces boulardii* Combined With Quadruple Therapy for *Helicobacter pylori* Eradication Decreased the Duration and Severity of Diarrhea: A Multi-Center Prospective Randomized Controlled Trial

**DOI:** 10.3389/fmed.2021.776955

**Published:** 2021-11-18

**Authors:** Yuchong Zhao, Yilei Yang, Aruna, Jun Xiao, Jun Song, Tizheng Huang, Shuyu Li, Jiguang Kou, Lu Huang, Dexiong Ji, Si Xiong, Wang Peng, Sanping Xu, Bin Cheng

**Affiliations:** ^1^Department of Gastroenterology and Hepatology, Tongji Hospital, Tongji Medical College, Huazhong University of Science and Technology, Wuhan, China; ^2^Department of Gastroenterology and Hepatology, Zhongnan Hospital of Wuhan University, Wuhan, China; ^3^Department of Gastroenterology and Hepatology, Union Hospital (West District), Tongji Medical College, Huazhong University of Science and Technology, Wuhan, China; ^4^Department of Gastroenterology and Hepatology, Hubei Aerospace Hospital, Xiaogan, China; ^5^Department of Gastroenterology and Hepatology, The Third People's Hospital of Hubei Province, Wuhan, China; ^6^Department of Gastroenterology and Hepatology, The Central Hospital of Xiaogan, Xiaogan, China; ^7^Department of Gastroenterology and Hepatology, The First People's Hospital of Xiaogan, Xiaogan, China; ^8^Department of Gastroenterology and Hepatology, Anlu People's Hospital, Anlu, China; ^9^Department of Gastroenterology and Hepatology, Union Hospital, Tongji Medical College, Huazhong University of Science and Technology, Wuhan, China

**Keywords:** *Helicobacter pylori*, *Saccharomyces boulardii*, eradication, diarrhea, quadruple therapy

## Abstract

**Background:** Whether probiotics helped the *Helicobacter pylori* (*H. pylori*) eradication was still highly controversial. The non-bacterial *Saccharomyces boulardii* (*S. boulardii*) has demonstrated its efficacy in the treatment of antibiotic-associated and infectious diarrhea. We aimed to evaluate the effects of *S. boulardii* combined with quadruple therapy for *H. pylori* eradication and associated side effects.

**Methods:** Three hundred and sixty *H. pylori-*infected patients were recruited in this multicenter, randomized controlled trial. The patients who underwent *H. pylori* eradication treatment were randomized in a ratio of 1:1 into two separate groups that received standard quadruple therapy (Group A) and quadruple therapy plus *S. boulardii* sachets (Group B) for 14 days. The everyday medication and side-effect records were collected for compliance and adverse effect analysis. All patients accepted ^13^C/^14^C-urea breath tests 4 weeks after the therapy completion.

**Results:** *Saccharomyces boulardii* and quadruple therapy-combined intervention significantly reduced the incidences of overall side effects (27.8 vs. 38.5%, *p* = 0.034) and diarrhea (11.2 vs. 21.2%, *p* = 0.012) in Group B compared with quadruple therapy alone in Group A, especially reduced the diarrhea duration (5.0 days vs. 7.7 days, *p* = 0.032) and incidence of severe diarrhea (4.7 vs. 10.1%, *p* = 0.040). Intention-to-treat (ITT) analysis and per-protocol (PP) analysis both indicated no statistical differences of eradication rate between Groups A and B (ITT: 82.7 vs. 85.8%, *p* = 0.426; PP: 89.7 vs. 94.2%, *p* = 0.146). The joint use of *S. boulardii* and quadruple therapy markedly improved the overall pre-eradication alimentary symptoms (hazard ratio (HR): 2.507, 95% CI: 1.449–4.338) recovery.

**Conclusion:** *Saccharomyces boulardii* ameliorated *H. pylori* eradication*-*induced antibiotic-associated side effects especially reduced the incidence of severe diarrhea and the duration of diarrhea. However, there was no significant effect of *S. boulardii* on the rate of *H. pylori* eradication.

**Trial Registration:** The protocol had retrospectively registered at ClinicalTrails.gov, Unique identifier: NCT03688828, date of registration: September 27, 2018; https://clinicaltrials.gov/show/NCT03688828

## Introduction

The prevalence of *Helicobacter pylori* (*H. pylori*) infection in the general population in China was approximately 60% (1, 2). The eradication of *H. pylori* has been demonstrated effective for alleviating various gastrointestinal (GI) diseases and reducing the risk of gastric cancer (3–6). Current Chinese guidelines recommended 14-day bismuth-containing quadruple therapy as a first-line regimen for *H. pylori* eradication (7). However, due to the increasing resistance to antibiotics and relatively high incidence of side effects, quadruple therapy was not as satisfying as before (8–10). Several previous studies indicated that the use of proton pump inhibitors (PPIs) and antibiotics led to dysbiosis and abundance changes of the gut microbiota (11–13). Though with a relatively low rate of severe side effects, the sporadic reports of *H. pylori* treatment-induced *Clostridium difficile* (*C. difficile*) infection and pseudomembranous colitis have been incessant over the past decades (14–18). Several novel regimens (e.g., high-dose PPI + amoxicillin dual therapy, and vonoprazan-containing therapy) were emerging to conquer the current difficulties, and the preliminary clinical data showed the effect of dual therapy was not inferior to quadruple therapy for *H. pylori* eradication whereas the efficacies of these regimens were restricted by poor compliance and availability (19–21). And the incidence of adverse events (AEs) was nearly equal between the novel treatment and quadruple therapy.

To prevent and treat the underlying AEs brought by *H. pylori* eradication, standard quadruple therapy with probiotic supplements, in particular, *lactobacilli, bifidobacterial*, and *Saccharomyces boulardii* (*S. boulardii*) were administrated (22). Non-pathogenic yeast *S. boulardii* was initially used to prevent the *C. difficile* infection and relapse, now it has demonstrated the efficacy of preventing and treating antibiotic-associated, infectious, and functional diarrhea (23, 24). The yeast nature of *S. boulardii* other than bacterial suggested the implications of joint use with antibiotics. Several reports claimed that *S. boulardii* protected intestinal epithelium against pathogen colonization and invasion through upregulating the secretion of sIgA into the luminal mucous, thus exerted the anti-*H. pylori* effect (25–27). However, current clinical studies to investigate the synergistic effect of *S. boulardii* on *H. pylori* infection were highly controversial, even some meta-analyses had contradictory results (28–33). And most of the current trials were about *S. boulardii* and triple therapy combination. Due to the high increasing resistance to antibiotics, triple therapy was no longer effective as used to be. Whether *S. boulardii* could improve the eradication rate of the highly effective 14-day quadruple therapy is still largely unknown.

Most clinical trials about *S. boulardii* focused on the synergetic effect on eradication, the evaluation of diarrhea prevention was usually the secondary aim. Antibiotic-associated diarrhea was the most common AE, ranged from 7.0 to 41.2% (33–38). Though most associated adverse effects were mild and tolerable, diarrhea was the main reason leading to eradication treatment discontinuation. To further investigate the diarrhea prevention and treatment effect of *S. boulardii* in *H. pylori* eradication therapy, we conducted this prospective multicenter randomized controlled trial. Meanwhile, we evaluated the potential synergistic effect of *S. boulardii* on *H. pylori* eradication and alimentary symptoms.

## Materials and Methods

### Patient Enrollment

This study was approved by the Ethical Committee of Tongji Hospital, Tongji Medical College, Huazhong University of Science and Technology (TJ-IRB20180904) and registered on ClinicalTrials.gov (NCT03688828). Between October 2018 and September 2019 from 9 medical centers in China, 360 patients between the ages of 22 and 65 years were enrolled in this study after receiving endoscopic evaluations for various GI symptoms. Each of these patients had ^13^C/^14^C-urea breath test proof of *H. pylori* infection. The exclusion criteria included (1) previous attempts to eradicate *H. pylori*; (2) pregnant or lactation; (3) hypersensitivity to the drugs being used in the study; (4) previous treatment with PPIs, bismuth, H2 receptor antagonist, or antibiotics within 4 weeks of the study; and (5) treatment with non-steroidal anti-inflammatory drugs (NSAIDs) or alcohol abuse during the study.

### Study Design

This was a randomized, parallel-group study. Three hundred and sixty *H. pylori*-infected patients were recruited in the study and randomly assigned by a computer program into two groups: standard quadruple therapy group (Group A) and quadruple therapy plus *S. bouladii* (Group B). Computer-generated randomization assignments were centralized using the block randomization method (block size of 8) by a data manager who was not involved in the data analysis or patient enrollment. Patients assigned to Group A received esomeprazole (AstraZeneca Pharmaceutical, Co. Ltd., Cambridge, United Kingdom) 20 mg two times a day (*bid*); amoxicillin (Baker Norton Pharmaceutical, Co., Ltd., Kunming, China) 1.0 g *bid*; clarithromycin (Abbott Laboratories Ltd., Shanghai, China) 500 mg bid; and Bismuth Potassium Citrate (Livzon Pharmaceutical Group, Inc., Zhuhai, China) 600 mg *bid* for 14 days. Patients assigned to Group B received the same quadruple therapy for 14 days, Additionally, *S. boulardii* sachets (Laboratories Biocodex, Inc., France) of 500 mg was given a bid to Group B for 14 days. Serious diarrhea patients (mushy stools or watery stools > two times a day) were additionally given montmorillonite powder 3 g *tid*. Considering the high resistance rate of metronidazole in the Center China population, we took amoxicillin and clarithromycin as our antibiotic choice (9, 39).

### Study Evaluations and Outcomes

**Figure 1:**
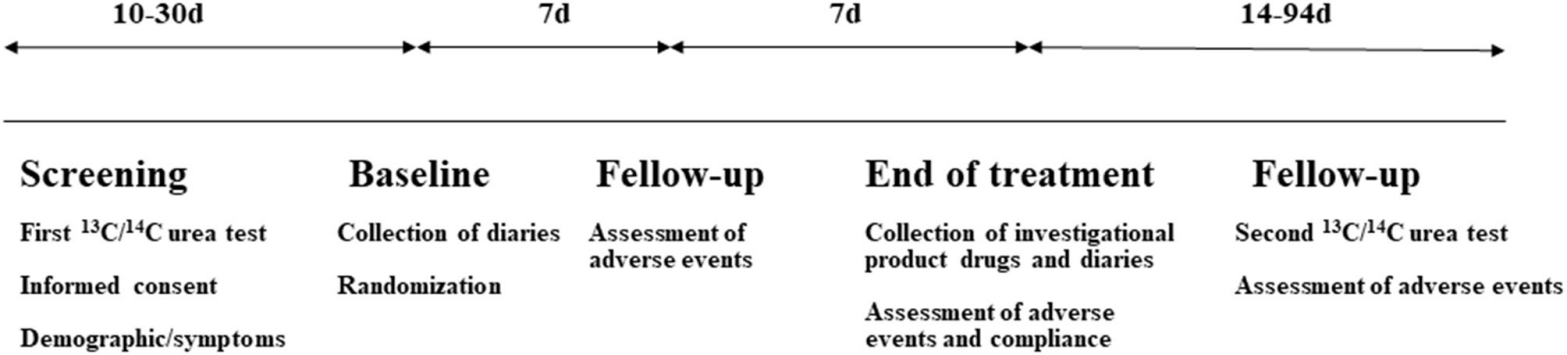
Flowchart of the study progress.

Patients were evaluated at five visits: screening (10–30 days before the baseline visit), baseline, 7 days after the treatment initiation, end of treatment/efficacy (14 days after the treatment initiation), and the second ^13^C/^14^C-urea breath test 4 weeks after the therapy completion and follow-up (44–94 days after treatment completion; Figure 1). ^13^C/^14^C-urea breath tests were applied to detect the *H. pylori* infection for the high sensitivity and specificity. Previous studies demonstrated that there were no statistical differences between the ^13^C and ^14^C-urea breath tests (40, 41). In this trial, 276 patients accepted the ^13^C-urea breath test and 84 patients received the ^14^C-urea breath test. The urea breath test technician was blinded to patient groups.

The primary outcome measure was the incidence of AEs. The investigator will record all AEs related to anti-*H. pylori* therapy, such as nausea, vomiting, taste abnormalities, abdominal pain, abdominal distension, diarrhea, and increased symptoms on the case report form. The incidence of adverse reactions will be assessed at three points: before treatment, during treatment (2 weeks), and after treatment (4–12 weeks). Patients returned their medication and side effects record form after the urea-test re-examination.

The secondary outcome measure was to investigate whether there was a statistical difference in the eradication rate between the two groups. Eradication rate = number of *H. pylori* eradicated cases after treatment/total cases ×100%. Non-ulcer patients will be tested 4 weeks after the end of the eradication treatment, and ulcer patients will be tested 2 weeks after the end of the total course of treatment. Eradication rates were determined by both ITT- and PP-based analyses. All enrolled patients were included in the ITT analysis, but the PP analysis excluded those patients who dropped out due to side effects, loss to follow-up, or poor compliance.

The effect of *S. boulardii* on *H. pylori* eradication rate and the incidence of AEs was also studied using binary logistic regression models, which included the following parameters: eradication rate, the overall incidence of AEs, and the incidence of antibiotic-induced diarrhea.

### Sample Size and Statistical Analysis

Based on a literature review of *H. pylori* eradication-induced antibiotic diarrhea (29, 35, 42), we expected a difference between quadruple therapy combined with *S. boulardii* and quadruple therapy alone on the incidence of diarrhea of 13.5 vs. 19.5%. The calculation yielded 179 for combined therapy and 179 for quadruple therapy, with a power of 80% and a two-sided significance level of 0.05 with an assumed 20% dropout rate. Each group should have 184 patients following the randomized block design of eight patients in a group. We calculated a final sample size of 368 patients (184 per group). The full analysis set should be as close as possible to the ITT set. The standards and population of the PP set will be finalized after data-blinding verification. The direct deletion method will be used to treat missing data.

In this study, the demographic and clinical characteristics of the patients will be summarized with mean and SD. The results of eradication and incidence of AEs are expressed in terms of the number of cases and percentage.

Qualitative variables were compared using the chi-squared test and Fisher's exact test, while Student's *t*-test and the Mann-Whitney *U* test were used for quantitative variables. The effect of *S. boulardii* plus sequential therapy on the eradication rate and the incidence of antibiotic-induced AEs were determined using binary logistic regression, and *p-*value < 0.05 with two-tail will be considered significant. All statistical analyses will be performed by blinded professional statisticians using SPSS V.26.0.

## Results

### Patient Disposition and Characteristics

**Figure 2:**
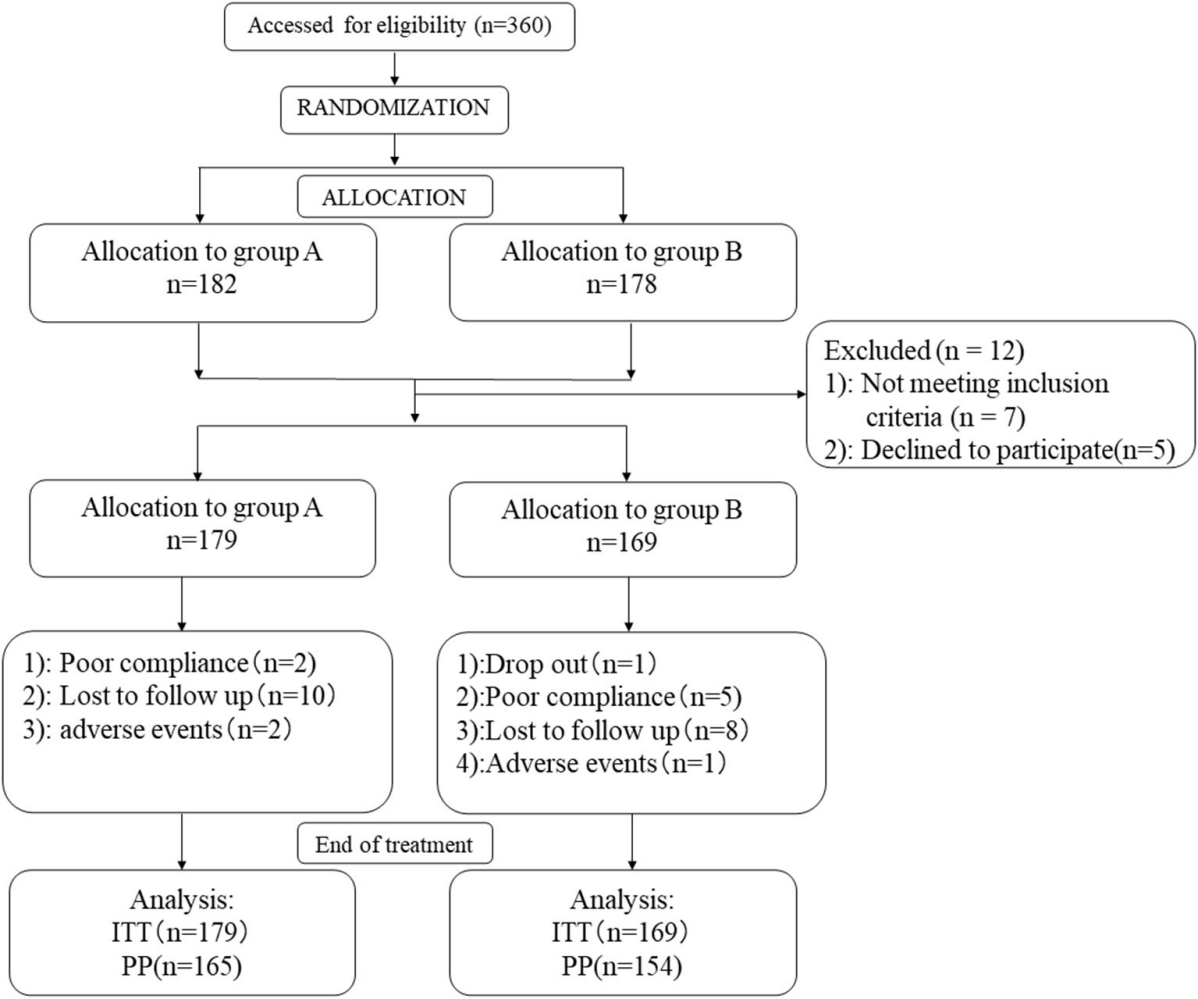
Profile of the study protocol.

A total of 348 patients fulfilling the inclusion criteria were enrolled in this trial, with 179 patients in Group A and 169 patients in Group B for ITT analysis. Twenty-nine patients (8.33%) were excluded from PP analysis. Follow-up was incomplete in 10 patients (5.6%) and 8 patients (4.7%) in Groups A and B, respectively. Two patients in Group A discontinued treatment because of severe diarrhea while one patient in Group B discontinued for skin rash. Poor treatment compliance was reported in two (1.1%) patients and five (3.0%) patients in Groups A and B. Apart from this, there was one patient who dropped out from treatment in group B because of pregnancy (Figure 2). At baseline, there were no statistically significant differences in the baseline characteristics of patients included in the two study groups (Table 1).

**Table 1:** Baseline characteristics of all patients.

**Characteristics**	**Group A**	**Group B**	***P*-value**
	**(*n* = 179)**	**(*n* = 169)**	
Age, mean ± SD, years	46.68 ± 12.85	45.31 ± 11.46	0.308
Sex, % of female	45.7%	49.1%	0.536
Endoscopic findings, *n* (%)	
Non-atrophic gastritis	47 (26.3%)	40 (23.7%)	0.577
Atrophic gastritis	21 (11.7%)	27 (16.0%)	0.251
Ulcer	32 (17.9%)	33 (19.5%)	0.693
Rural/urban, % of rural	70.4%	68.0%	0.636
Pre-treatment symptoms, *n* (%)	127 (75.1%)	126 (70.4%)	0.321
Epigastric pain	63 (37.3%)	54 (30.2%)	0.161
Epigastric distending	66 (39.1%)	66 (36.9%)	0.676
Regurgitation/heartburn	34 (20.1%)	43 (25.4%)	0.382
Frequent belching	29 (17.2%)	20 (11.2%)	0.109
Nausea/vomiting	19 (11.2%)	15 (8.4%)	0.370

### Efficacy Analysis

Intention-to-treat analysis demonstrated that the eradication rates were 85.8% for Group B and 82.7% for Group A (hazard ratio, HR, = 1.038, *p* = 0.426, 95% CI = 0.948–1.136). PP analysis indicated that the eradication results were 89.7% for Group B and 94.2% for Group A (HR = 1.851, *p* = 0.146, 95% CI = 0.799–4.286; Table 2). Both ITT and PP analyses showed no statistical differences in the eradication rate between Groups A and B.

**Table 2:** Comparison of clinical therapeutic effect analysis between two groups.

**Therapeutic effect**	**Group A**	**Group B**	***P*-value**
Eradication rate			
ITT	148/179 (82.7%)	145/169 (85.8%)	0.426
PP	148/165 (89.7%)	145/154 (94.2%)	0.146
3-month symptom complete relief rate	74/127 (58.3%)	98/126 (78.6%)	<0.001^***^
Epigastric pain	53/63 (81.5%)	50/54 (75.8%)	0.162
Abdominal distension	35/66 (53.0%)	49/66 (89.4%)	0.011^*^
REGURGITATION/heartburn	26/34 (76.5%)	35/43 (81.4%)	0.603
Frequent belching	21/29 (72.4%)	16/20 (80.0%)	0.122
Nausea/vomiting	14/19 (74.7%)	13/15 (86.7%)	0.368

*ITT, intention-to-treat analysis; PP, per-protocol analysis*.

* *p < 0.05, significant difference*,

*** *p < 0.001*.

The follow-up analysis of the alimentary symptoms, from pre-treatment of 3 months after the eradication, indicated that the overall symptoms improvement rate of Group B (78.6 vs. 58.3%, *p* < 0.001) was significantly higher than that of Group A (Table 2). Further analysis showed that the abdominal distension recovery rate in Group B compared was markedly higher than in Group A (89.4 vs. 53.0%, *p* = 0.011).

### Side Effects Analysis

**Figure 3:**
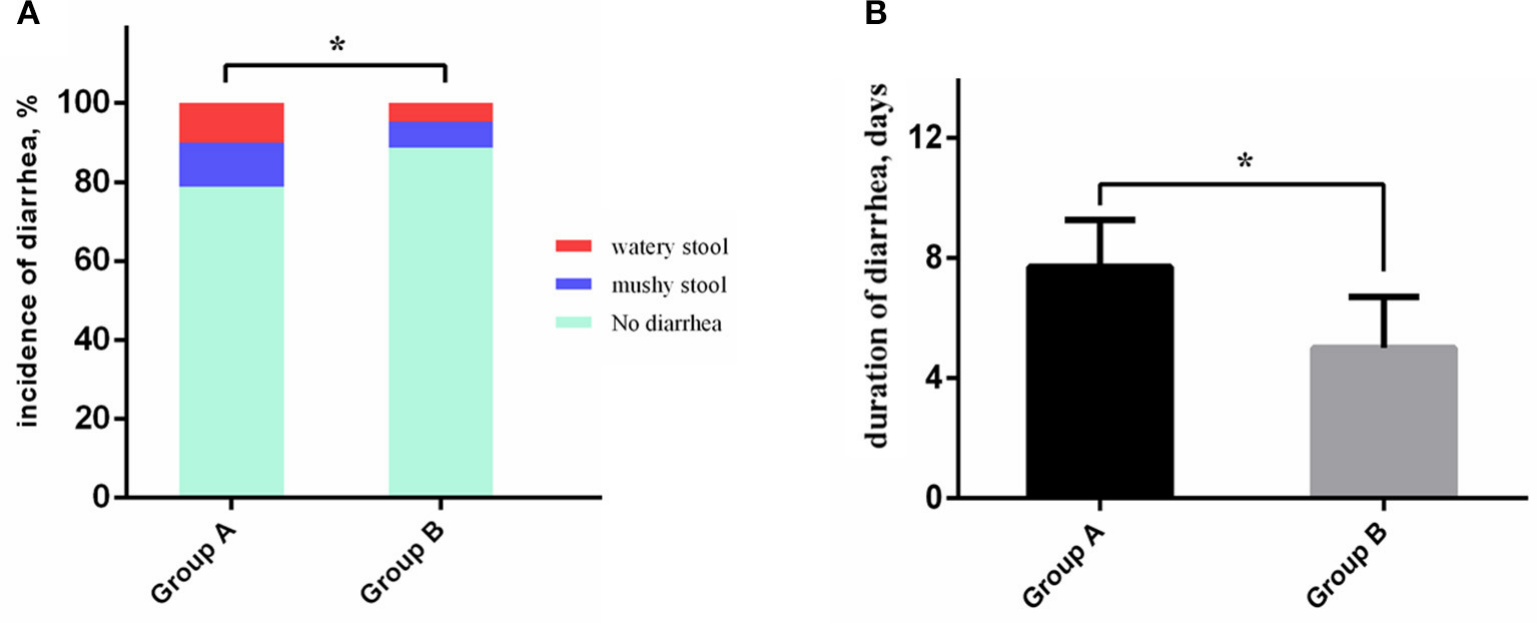
Antibiotic-associated diarrhea characteristics comparison between the two groups. **(A)** The severe diarrhea patients and overall diarrhea patients occupy a bigger proportion in Group A than in Group B. **(B)** The duration of diarrhea of patients in Group B was significantly shorter than in Group A. *P < 0.05.

The overall incidences of AEs in the experimental and control groups were 38.5 and 27.8%, respectively, representing a decrease of 10.7% in the experimental group (*p* = 0.034). The diarrhea rate of Group A was significantly higher than that of Group B (21.2 vs. 11.2%, *p* = 0.012). Meanwhile, the combination of *S. boulardii* and quadruple therapy decreased the duration of diarrhea (5.0 days vs. 7.7 days, *p* = 0.032) and incidence of severe diarrhea (10.1 vs. 4.7%, *p* = 0.040) in Group B compared with Group A (Figure 3). There were no statistical differences between Groups A and B in terms of vomiting, constipation, or allergy (Table 3). Results of the multivariate analysis further verified that the combination of *S. boulardii* and quadruple treatment reduced the overall incidence of AEs (odds ratio, OR: 0.378, 95% CI: 0.117–0.807) and incidence of diarrhea (OR: 0.359, 95% CI*:* 0.148–0.872) compared with quadruple therapy alone (Table 4). Two patients in Group A accepted intravenous (*i.v*.) treatment for severe diarrhea other than montmorillonite powder and discontinued the eradication therapy. However, no *C. difficile* was detected by fecal examination. And no patients in Group B discontinued the therapy due to diarrhea.

**Table 3:** Comparison of incidence of AEs between two groups.

**Adverse events**	**Group A**	**Group B**	***P*-value**
Overall adverse events, n (%)	69 (38.5%)	47 (27.8%)	0.034^*^
Vomiting	20 (11.2%)	27 (15.9%)	0.190
Diarrhea	38 (21.2%)	19 (11.2%)	0.012^*^
Duration of diarrhea ± SEM, days	7.7 (0.7)	5.0 (0.8)	0.032^*^
Watery stools	18 (10.1%)	8 (4.7%)	0.040^*^
Mushy stools	20 (11.2%)	11 (6.5%)	
Allergy	3 (1.7%)	1 (0.6%)	0.623
Constipation	6 (3.4%)	5 (3.0%)	0.834

*SEM, standard error of mean; AEs, adverse events*.

* *p < 0.05, significant difference*.

**Table 4:** Comparison of incidence of AEs among two groups.

**Model**	**Group A**		**Group B**	
	**OR (95% CI)**	***P*-value**	**OR (95% CI)**	***P*-value**
Incidence of overall AE				
Crude model	1.000 (*ref*.)	-	0.614 (0.391–0.965)	0.034^*^
Model 1	1.000 (*ref*.)	-	0.643 (0.399–1.006)	0.053
Model 2	1.000 (*ref*.)	-	0.378 (0.117–0.807)	0.012^*^
Incidence of diarrhea				
Crude model	1.000 (*ref*.)	-	0.470 (0.259–0.854)	0.013^*^
Model 1	1.000 (*ref*.)	-	0.486 (0.265–0.893)	0.020^*^
Model 2	1.000 (*ref*.)	-	0.359 (0.148–0.872)	0.024^*^

*Statisitical analyses are based on a multivariable logistic regression model. AEs, adverse events. Model 1 was adjusted for age and sex. Model 2 was additionally adjusted for rural/urban, endoscopic features, and different medical centers*.

* *p < 0.05, significant difference*.

## Discussion

The synergetic effect of *S. boulardii* on *H. pylori* eradication and associated side effects were analyzed in this study through the first multicenter randomized controlled trials of *S. boulardii* and quadruple therapy combination in China. We demonstrated that the administration of *S. boulardii* significantly decreased the incidence of eradication-associated AEs (OR: 0.378, 95% CI: 0.117–0.807), especially reduced the duration of diarrhea (5.0 days vs. 7.7 days, *p* = 0.032) and incidence of severe diarrhea (4.7 vs. 10.1%, *p* = 0.040). However, *S. boulardii* did not improve the eradication rate for bismuth quadruple therapy. However, the joint use of *S. boulardii* significantly improved gastritis/ulcer-associated symptoms (HR: 2.507, 95% CI: 1.449–4.338).

Unlike synergetic effects of *H. pylori* eradication, the effect of probiotics on AEs prevention and treatment was definite. Distinct from other bacterial probiotics, *S. boulardii* is a non-pathogenic fungus resistant to gastric acid and antibiotics, thus it could be used with eradication therapy simultaneously (24, 25). *S. boulardii* was used for the prevention of *C. difficile* infection originally (43). With sporadic reports of pseudomembranous colitis during *H. pylori* eradication, *S. boulardii* was gradually used as a supplement for the prevention and treatment of AEs. Acute severe diarrhea was the main reason for eradication therapy discontinuation thus leading to treatment failure. And some reports claimed that the diarrhea prevention effect of *S. boulardii* was only notable in children but not in adults (35, 44). Although various trials have already demonstrated the efficacy of *S. boulardii* to prevent and treat antibiotic-associated diarrhea (33, 35, 45, 46). Our study refined the diarrhea-associated data and verified that *S. boulardii* reduced the diarrhea duration and incidence of severe diarrhea correlated with eradication therapy. None of the diarrhea patients in the quadruple therapy plus *S. boulardii* group required further diarrhea treatment, whereas two patients in the quadruple therapy alone group accepted intravenous fluid infusion.

Probiotics might be effective for improving *H. pylori* eradication rates due to the decrease in the AEs and potential mucosal barrier protective effect (29, 36, 37, 47). Previous studies demonstrated the probiotics exerted the synergetic eradication effect through a similar mechanism including competitively inhibiting the *H. pylori* adhesion to gastric mucosa (48, 49) or producing antimicrobial molecules (29, 50). Recently, Yang et al. reported that the administration of *S. boulardii* could inhibit the *H. pylori* infection-induced gastric lymphoid follicle formation (26). Previous experiments mainly adopted the combination of *S. boulardii* and triple therapy. However, due to high resistance and decreasing efficacy, triple therapy was no longer the first-line recommendation for *H. pylori* eradication in China (7). The eradication rate for bismuth quadruple therapy has already reached 87.6–92.6% (51). In addition, the synergetic effect of *S. boulardii* on eradication was highly controversial for many years even with low-efficacy triple therapy. Some meta-analyses gave contradictory results, let alone some prospective and respective trials. Szayewska et al. reported in a meta-analysis that *S. boulardii* improved the eradication rate [Risk Ratio (RR): 1.11, 95% CI: 1.06–1.17; moderate-quality evidence] whereas Wang et al. found no better efficacy of any probiotic supplement for *H. pylori* eradication (24, 29). This is the first multicenter randomized controlled trial for *S. boulardii* and bismuth quadruple therapy in China. And our study showed that this probiotic supplement barely improved the eradication rate of quadruple therapy. Considering the eradication rate was still at a satisfying level compared with triple therapy, no improvement after the *S. boulardii* supplement became reasonable. It was a novel finding that joint administration of *S. boulardii* significantly improved the original dyspepsia symptoms.

Collectively, our study demonstrated that *S. boulardii*-ameliorated *H. pylori* eradication-induced antibiotic-associated diarrhea especially decreased the diarrhea duration and the incidence of severe diarrhea. Different from the combination with triple therapy, *S. boulardii* did not affect the quadruple therapy eradication rate of *H. pylori*, whereas improved the pre-treatment dyspepsia symptoms.

## Data Availability Statement

The raw data supporting the conclusions of this article will be made available by the authors, without undue reservation.

## Ethics Statement

The studies involving human participants were reviewed and approved by the Ethical Committee of Tongji Hospital, Tongji Medical College, Huazhong University of Science and Technology. The patients/participants provided their written informed consent to participate in this study.

## Author Contributions

BC and SXu designed the study. YZ and YY performed data analysis. SXi, YZ, A, JX, SL, JK, TH, LH, and DJ performed the acquisition of data. YZ and YY drafted the manuscript. SXi provided critical revision of the manuscript for important intellectual content. YZ, SXi, and YY performed technical support. BC and SXu performed study supervision. All authors have read and approved the manuscript.

## Funding

This work was supported by the National Natural Science Foundation of China Grant Nos. 81802427 (to ZL), 81372352 (to BC).

## Conflict of Interest

The authors declare that the research was conducted in the absence of any commercial or financial relationships that could be construed as a potential conflict of interest.

## Publisher's Note

All claims expressed in this article are solely those of the authors and do not necessarily represent those of their affiliated organizations, or those of the publisher, the editors and the reviewers. Any product that may be evaluated in this article, or claim that may be made by its manufacturer, is not guaranteed or endorsed by the publisher.

## Abbreviations

PPI: proton pump inhibitors
GI: gastrointestinal
AEs: adverse events
PP: Per-protocol
ITT: Intention-to-treat
*H. pylori*: *Helicobacter pylori*
*S. boulardii, Saccharomyces boulardii; C. difficile*: *Clostridium difficile*.
